# Enrichment of rare methanogenic Archaea shows their important ecological role in natural high-CO_2_ terrestrial subsurface environments

**DOI:** 10.3389/fmicb.2023.1105259

**Published:** 2023-05-24

**Authors:** Zeyu Jia, Daniel Lipus, Oliver Burckhardt, Robert Bussert, Megan Sondermann, Alexander Bartholomäus, Dirk Wagner, Jens Kallmeyer

**Affiliations:** ^1^GFZ German Research Centre for Geosciences, Section Geomicrobiology, Potsdam, Germany; ^2^Applied Geochemistry, Institute of Applied Geosciences, Technische Universität Berlin, Berlin, Germany; ^3^Institute of Geosciences, University of Potsdam, Potsdam, Germany

**Keywords:** Archaea, methanogens, deep biosphere, CCS - carbon capture and sequestration, enrichment culture

## Abstract

**Introduction:**

Long-term stability of underground CO_2_ storage is partially affected by microbial activity but our knowledge of these effects is limited, mainly due to a lack of sites. A consistently high flux of mantle-derived CO_2_ makes the Eger Rift in the Czech Republic a natural analogue to underground CO_2_ storage. The Eger Rift is a seismically active region and H_2_ is produced abiotically during earthquakes, providing energy to indigenous microbial communities.

**Methods:**

To investigate the response of a microbial ecosystem to high levels of CO_2_ and H_2_, we enriched microorganisms from samples from a 239.5 m long drill core from the Eger Rift. Microbial abundance, diversity and community structure were assessed using qPCR and 16S rRNA gene sequencing. Enrichment cultures were set up with minimal mineral media and H_2_/CO_2_ headspace to simulate a seismically active period with elevated H_2_.

**Results and discussion:**

Methane headspace concentrations in the enrichments indicated that active methanogens were almost exclusively restricted to enrichment cultures from Miocene lacustrine deposits (50–60 m), for which we observed the most significant growth. Taxonomic assessment showed microbial communities in these enrichments to be less diverse than those with little or no growth. Active enrichments were especially abundant in methanogens of the taxa *Methanobacterium* and *Methanosphaerula*. Concurrent to the emergence of methanogenic archaea, we also observed sulfate reducers with the metabolic ability to utilize H_2_ and CO_2_, specifically the genus *Desulfosporosinus*, which were able to outcompete methanogens in several enrichments. Low microbial abundance and a diverse non-CO_2_ driven microbial community, similar to that in drill core samples, also reflect the inactivity in these cultures. Significant growth of sulfate reducing and methanogenic microbial taxa, which make up only a small fraction of the total microbial community, emphasize the need to account for rare biosphere taxa when assessing the metabolic potential of microbial subsurface populations. The observation that CO_2_ and H_2_-utilizing microorganisms could only be enriched from a narrow depth interval suggests that factors such as sediment heterogeneity may also be important. This study provides new insight on subsurface microbes under the influence of high CO_2_ concentrations, similar to those found in CCS sites.

## Introduction

Growing concerns about global warming due to increasing atmospheric CO_2_ concentrations bring attention towards Carbon capture and storage (CCS), an engineering solution to limit the release of CO_2_ into the atmosphere by capturing the CO_2_ at its source and storing it in suitable underground reservoirs, i.e., deep saline aquifers or depleted hydrocarbon reservoirs. Demonstration projects such as CO2SINK in Ketzin, Germany and FutureGen in Illinois, United States provide scientists the opportunity to study the impact of CCS on the surrounding environment, including microbial communities living in these ecosystems (Morozova et al., 2011; Gilmore et al., 2014; Pellizzari et al., 2016). However, until now the majority of CCS facilities have only been operated for a relatively short time period, leaving the long-term effects of elevated CO_2_ concentrations in subsurface environments and thus long-term stability of CCS largely unclear. Natural subsurface high-CO_2_ environments could serve as an analog to study the long-term effects of high CO_2_ concentrations on subsurface ecosystems.

The Eger Rift, located in West Bohemia (Czech Republic), is characterized by active mantle degassing of CO_2_ and thus serves as a natural analog site for CCS. It’s reasonable to assume that degassing has been ongoing since the mid-Pleistocene (Weinlich et al., 1999), releasing high amounts of CO_2_ for roughly 1 million years. Measurements of gas emissions at one of the known Eger Rift Mofette fields in Hartusov varied between ∼ 14 and 43 kg m^−2^ d^−1^ (Nickschick et al., 2015). In order to safely store CO_2_ in a subsurface reservoir there has to be an impermeable geologic seal that prevents the CO_2_ from ascending. Typical geologic seals are impermeable clays or claystones, or evaporites. The Hartusov Mofette field features such a seal, in this case palustrine claystone (Main Seam Fm.) that overlay lacustrine sandstones (Cypris Fm.), thus forming a contained subsurface CO_2_ reservoir (Bussert et al., 2017). Groundwater from this section was determined to be highly gas-saturated with about 2 g L^−1^ of free dissolved CO_2_. Thus, this location provides a natural analogue of a CCS storage site, allowing a glimpse into the long-term development of microbial communities under high CO_2_ conditions. We therefore assume that microbial life in this peculiar natural system has likely reached steady state with regard to adaptation to high CO_2_ conditions that may therefore serve as an example for future microbial communities in and around CCS sites after many years of operation.

Investigating the impact of CO_2_ on subsurface microbial communities is of significant scientific and societal interest, as elevated concentrations of CO_2_ can influence and disturb microbial communities on several levels. Firstly, the dissolution of CO_2_ in water decreases the pH, contributing to the increase of acidotolerant and acidophilic microbes in the community (Krüger et al., 2011; Beulig et al., 2015). Secondly, studies at biochemical and cellular aspects show negative or even lethal effects of high concentration of CO_2_ on microbial cells, including the damage of cell membrane and changes in enzyme activities (Burrell et al., 2015; Wan et al., 2016). These negative effects could result in decrease of community abundance and diversity, along with a shift in community structure (Sáenz de Miera et al., 2014; Liu et al., 2018). Thirdly, CO_2_ also represents a bioavailable carbon source and potentially supporting the growth of autotrophs (Bornemann et al., 2022).

Similarly, hydrogen is a substrate and potential electron donor for chemolithotrophic growth of subsurface microbes (Osburn et al., 2014; Gregory et al., 2019). The presence of H_2_ in subsurface settings can be driven by several abiotic and biotic processes. Examples of abiogenic process generating H_2_ are graphitization, i.e., splitting methane into graphite, which occurs at temperatures above 600°C, the alteration of igneous ferromagnesium minerals at elevated pressure (35 MPa) and temperature around 300°C, the cataclasis of radicals exposed on the surface of crushed rock reacting with water to produce H_2_, or the radiolysis of water by radioactive uranium, thorium, potassium, etc. While the *in-situ* conditions of these processes can be unfavorable or lethal for microbial growth, the produced H_2_ could migrate and ascend to more microbe-friendly environments, becoming a substrate for microbial life. H_2_ could also be produced from several biochemical reactions that involve the enzyme hydrogenase, such as fermentation, nitrogen fixation, CO oxidation, or acetate oxidation (Gregory et al., 2019). In the Eger Rift, the occurrence of appreciable concentrations of H_2_ has been closely linked to earthquake swarms (Bräuer et al., 2008), suggesting a geogenic origin of the H_2_ through the cataclasis process mentioned above. There is experimental evidence that mechano-chemical reactions between groundwater and crushed rock can generate H_2_ (Kita et al., 1982), providing a potential explanation for the occurrence of H_2_ during earthquake swarms. In springs located in the Eger Rift region biogenic methane concentrations increased following the rise in H_2_ concentrations during earthquake swarms (Bräuer et al., 2005, 2014), providing evidence for methanogenic activity in the subsurface of Eger Rift. This phenomenon has also been observed in lab settings before, as Parkes et al. (2019) reported that a wide range of crushed rock (such as basalt, granite and silica) supplied enough H_2_ to support the growth of H_2_-utilizing methanogens.

Both CO_2_ and H_2_ may promote the growth of chemolithotrophic microorganisms, as the presence of both these substrates creates a particularly favorable environment for hydrogenotrophic methanogens. In addition, other groups of microorganisms may also compete for the available H_2_. A potential consumer of H_2_ and thus a competitor to methanogens are lithotrophic sulfate reducing bacteria (SRB) (Muyzer and Stams, 2008), even if sulfate is not present. This group of microorganisms can harvest energy from the reduction of sulfate or other electron acceptors to fix CO_2_. Both methanogenic archaea and sulfate reducing bacteria, especially when abundant and active, can contribute to the production of undesirable gasses (CH_4_ and H_2_S) with the latter potentially responsible for corrosion and biofouling events. To avoid deleterious microbial processes, which may directly or indirectly affect operations of CCS facilities it is necessary to study microbial populations and their potential roles. Therefore, detailed examinations focusing on methanogenic and other CO_2_ utilizing microbial populations in underground CO_2_ reservoirs are a key piece when investigating the impact of CCS on subsurface ecosystems.

**Figure 1:**
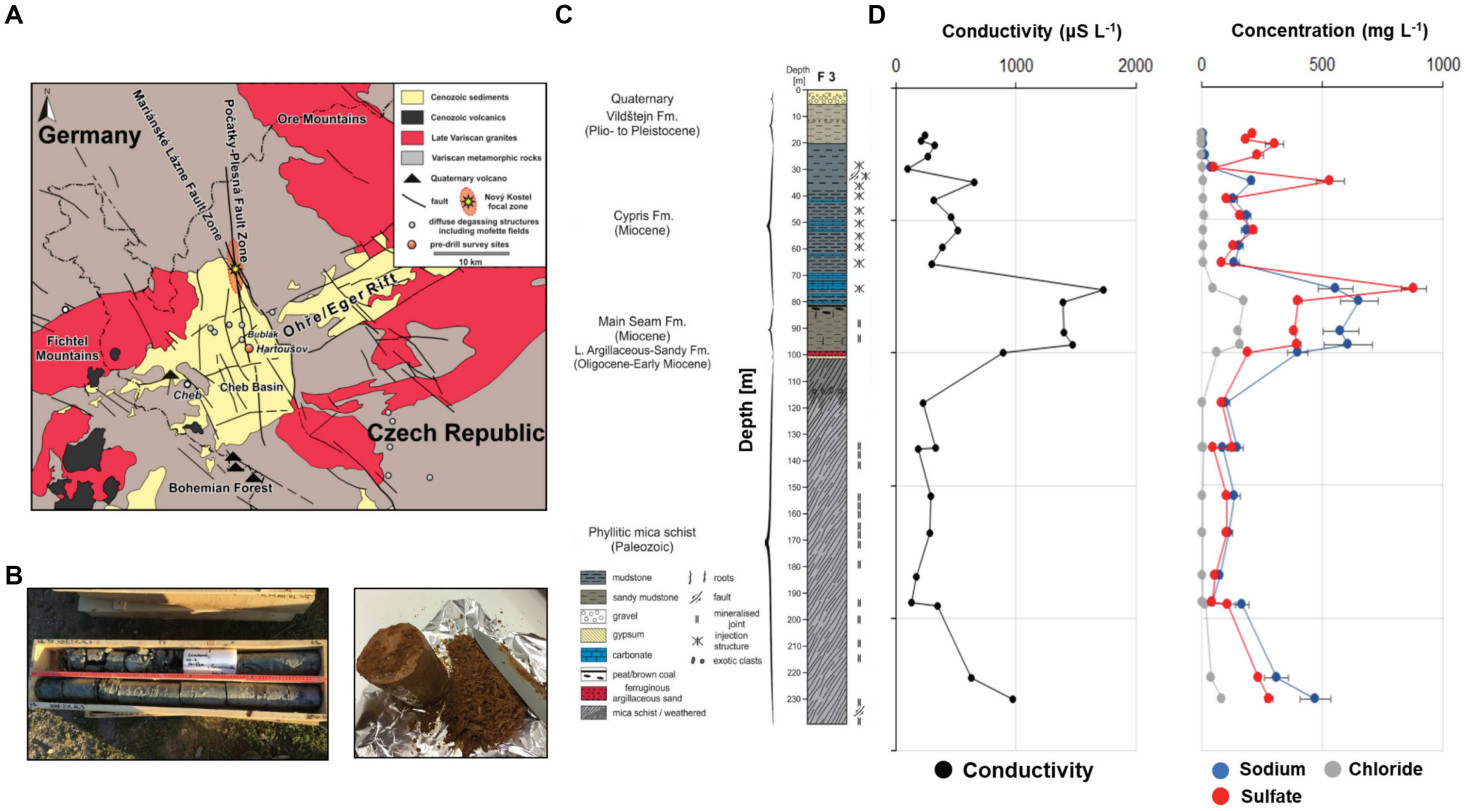
Map depicting the location of the Eger Rift the drill core was recovered from **(A)**, pictures of drill core segments used for enrichment experiments **(B)**, core description of the recovered drill core sediments **(C)**, geochemical profile showing conductivity and sodium, chloride and sulfate concentrations obtained through ion leeching (D).

In an effort to address the role of methanogens and other microorganisms that may play an important role in CCS facilities and impact their long-term functioning, this study evaluates the potential of methanogenesis in a natural, underground CO_2_ reservoir. We used sediment and rock samples from a recent drilling campaign in the Hartoušov Mofette Field (HMF) the Eger Rift, West Bohemia, Czech Republic. Detailed geological, geochemical and microbial assessments of sediments from this environment are currently available elsewhere (Lipus et al., 2022). Most important findings from this baseline data are also reported below (e.g., Figure 1D) providing the foundation for the incubation experiments performed in this study. Here, we are building on this data by specifically targeting microbial communities utilizing the available geogenic CO_2_ and H_2_. Using drill core samples collected from depths between 30 m to 230 m below surface we set up anaerobic enrichment cultures with H_2_/CO_2_ headspace with monitored methanogenic activity. Structure and function of the enriched communities were investigated through high-throughput sequencing of the 16S rRNA gene and quantitative PCR. This work aims (1) to confirm in the laboratory the on-site observation of biological methanogenesis proposed by Brauer et al. and later confirmed through isotopic analysis (Bräuer et al., 2005, 2014), particularly upon increased hydrogen availability and (2) to investigate the impact of geological disturbance on microbial communities, and (3) to identify active organisms that show a positive response to these disturbances.

## Materials and methods

### Site description and sampling

Drilling was conducted in August 2019 at the Hartoušov Mofette field in the Cheb Basin in the Western part of the Eger Rift (Figure 1A) as part of the “Drilling the Eger Rift” project of the International Continental Scientific Drilling Program (ICDP). The drill site F3 was approximately 60 m away from an active CO_2_ vent, with a gas composition of more than 99% of CO_2_ and emission rates of up to 125 kg m^−2^ d^−1^ (Nickschick et al., 2015). In the vicinity to this drill site, two exploratory wells (F1 and F2) had been drilled in 2007 and 2016, reaching 28 m and 108 m, respectively.

The details of drilling and subsampling procedures can be found in Lipus et al. (2022). Briefly, we reached a maximum depth of 238 m and recovered 163.15 m of core material (82.3% recovery rate). Possible contamination of the core through infiltration of drill mud was monitored *via* the addition of a fluorescent particle tracer (Friese et al., 2017; Kallmeyer, 2017). Tracer was added periodically to the drill mud and monitored to maintain a sufficiently high concentration of at least 10^8^ particles mL^−1^, which is at least three orders of magnitude higher than the microbial cell abundance in the drill mud.

Drill core sections (Figure 1B) were subsampled on site immediately after recovery to minimize contamination, exposure to oxygen and maintain the original microbial community. All core samples had the drill mud contaminated rim removed on site (~1 cm). Samples for downstream geochemical and culturing experiments were then sectioned into 10 cm whole round cores and placed in CO_2_-flushed gas-tight aluminum foil bags and stored at 4°C. Samples for molecular biology analysis were also sectioned, placed in gas-tight aluminum foil bags, and immediately frozen in liquid nitrogen. After transport to the Deutsche Geoforschungszentrum GFZ in Potsdam, geochemical and culturing samples were kept at 4°C, while those for molecular biology analysis were transferred to -80°C freezers. Ionic composition of the pore water of the drill core samples is described in Lipus et al. (2022).

After collection of the samples for microbiological and geochemical analyses, the remaining core sections were logged, cut into 1 m pieces, labeled, stored in wooden core boxes and transported to the “Nationales Bohrkernlager für kontinentale Forschungsbohrungen” in Berlin for detailed core description and long-term storage. The recovered core can be divided into six lithostratigraphic units (Figure 1C), with the lowermost sections (239.5 m to ~100.0 m) consisting of phyllitic mica schists and belonging to the Lower Paleozoic Saxothuringian basement of the Cheb Basin. The basement rocks, are overlain by clayey-silty sandstone of the Oligocene to Early Miocene Lower Argillaceous-Sandy Fm. (~100.00 m to ~98.36 m), massive to weakly stratified gray to brown and frequently mottled sandy to peaty mudstones of the Early Miocene Main Seam Fm (~98.36 m to ~80.50 m) consisting mostly of gray to green mudstones, followed by the Miocene Cypris Fm. (~80.5- ~20 m). Surface sediments consist mainly of green to gray, moderately stratified to massive sandy clay (~20 m to ~5.35 m) of the Plio- to Pleistocene Vildštejn Fm., and moderately to poorly sorted clayey sand and gravel (~5.35 m to surface), most probably representing Quaternary channel and floodplain deposits of the nearby Plesná River. A detailed core description can be found in Lipus et al. (2022).

### Anaerobic enrichment

We took subsamples for cultivation from the drill core samples stored at +4°C in CO_2_-flushed foil bags. Subsampling was carried out inside an anaerobic glove box (Whitley MG500 anaerobic workstation). We chose samples from 7 different depths (30, 35, 54, 60, 109, 136, and 222 m) to cover most of the depth range of the drill core and all lithological units except the uppermost one. Selection of these samples was based on preliminary enrichment data and the pristine state of the sampling containers. Sediment used for enrichments was only collected from the inner section of the whole round core. Five grams of sample were weighed and transferred to an autoclaved serum bottle. Quadruplicates were prepared for samples from each depth and named A, B, C or D, respectively. The bottles were then sealed again and flushed with N_2_/CO_2_ gas.

The medium used for anaerobic enrichments was based on a modified version of anaerobic medium (Balch et al., 1979). For 1 l medium, 10 mL solution A (10% NH_4_Cl, 10% MgCl_2_ · 6H_2_O, 4% CaCl_2_ · 2H_2_O), 10 ml trace elements solution (50 ppm EDTA, 15 ppm CoCl_2_ · 6H_2_O, 10 ppm MnCl_2_ · 4H_2_O, 10 ppm FeSO_4_ · 7H_2_O, 10 ppm ZnCl_2_, 3 ppm Na_2_WO_4_ · 2H_2_O, 3 ppm NiCl_2_ · 6H_2_O, 2 ppm Na_2_SeO_3_ · 5H_2_O, 3.45 ppm CuCl_2_ · 2 H_2_O, 1 ppm H_3_BO_3_, 1 ppm Na_2_MoO_4_ · 2 H_2_O), 2 mL 0.1% Resazurin solution was mixed with 864 ml MilliQ H_2_O in a 2-Liter anaerobic medium flask and autoclaved. The solution was cooled while bubbling with of N_2_/CO_2_ (20:80% ratio) gas, and 50 mL filter-sterilized 16% NaHCO_3_ solution, 30 mL autoclaved 10 mM cysteine solution, 2 mL autoclaved 28.76% KH_2_PO_4_ solution, and 10 mL filter-sterilized vitamin mixture solution (1 ppm Vitamin B10, 1 ppm nicotine acid, 1 ppm Calcium pantothenate, 1 ppm Vitamin B6, 1 ppm Riboflavin, 1 ppm thiamine-hydrochloride, 0.5 ppm biotin, 0.5 ppm folic acid, 0.5 ppm lipoic acid, 0.5 ppm Vitamin B12) were added. The pH of the medium was adjusted to 7.2. 20 mL of medium was then dispensed into each serum bottle containing a sediment sample. Finally, bottles were flushed with a H_2_/CO_2_ gas mixture (20:80% ratio) to replace the headspace with 3 rounds of vacuum and refill. Samples were incubated at 22°C in the dark. Enrichment cultures were named according to core depth and replicate, e.g., “30A” for the first replicate of a sample from 30 m depth. We replaced the headspace gas on day 49 for sample 60B, on day 71 for sample 54C, 54D, 60B, 60C and on day 96 for all the replicates.

### Headspace CH_4_ measurements

We monitored the production of methane in the headspace of the enrichment vials in approximately 1-week intervals using gas chromatography. Methane concentration was measured using an Agilent 7890A GC System equipped with a flame ionization detector and Agilent HP PLOT Q column under standard curve mode. The program was set as follow: inlet heater at 180°C, 18.733 psi steady pressure, 3 ml min^−1^ septum purge flow; column flow at 15 mL min^−1^ at 100°C for 1.5 min. We calibrated the standard curve with two 250 μL analytic pure standard samples with methane concentrations of 10 and 5,000 ppmv. Roughly 300 μL headspace gas was collected with a glass syringe from the enrichment cultures. Excessive gas was pushed out until a final volume of 250 μL was reached shortly before we loaded the gas sample into the GC.

### DNA extraction

Enrichments were subsampled for DNA extraction under anaerobic conditions after 96 days of incubation. 1 mL of enrichment slurry was transferred to a 2 ml Eppendorf tube, and immediately preserved at -80°C until further processing. DNA was extracted using Qiagen DNeasy Power Soil pro kit (Qiagen, Venlo, Netherlands) following the manufacturer’s protocol. Negative controls were included in every round of extraction. DNA concentrations were assessed using Qubit (Life Technologies, Carlsbad, CA, United States) and an Agilent tape station (Agilent, Santa Clara, CA, United States).

### Polymerase chain reaction (PCR) and Illumina sequencing

The V4 region of the 16S rRNA gene was amplified using 515F and 806R (Supplementary Table S1) barcoded primers targeting both Bacteria and Archaea with *in silico* coverages higher than 96% for both Bacteria and Archaea (Wasimuddin Schlaeppi et al., 2020). PCR reactions were run for 35 cycles (30s at 95°C, 45 s at 56°C, 60s at 72°C). Extraction controls and template (PCR) controls were included in each PCR run using their own set of barcoded primers. *E. coli* genomic DNA was included as positive control. PCR products were then pooled and cleaned using AMPure XP beads (Beckman Coulter, Pasadena, CA, United States). Concentration of PCR products was assessed using Qubit technology (Life Technologies, Carlsbad, CA, United States) and pooled in equimolar amounts at 30 ng per sample at most. The pooled DNA library was concentrated with an Eppendorf Concentrator plus (Eppendorf AG, Hamburg, Germany) and sequenced on an Illumina Miseq Sequencer by Eurofins Genomics (Ebersberg, Germany).

### Quantitative PCR

The quantification of total bacterial 16S rRNA gene and total archaeal 16S rRNA gene were carried out on BIO-RAD CFX Connect Real-Time System (Bio-Rad Laboratories, California, United States) with primer Eub341-F/Eub534-R, Parch340-F/Arch1000-R, respectively. Each reaction consists of 10 μL 2x SensiFAST SYBR mix, 0.08 μL 100 μM forward and backward primers each, 5.84 μL PCR water and 4 μl of template. The qPCR programs for each used primer combinations were similar: 95°C for 3 min, followed with 40 cycles of 3 s at 95°C, 20 s annealing at 60°C, 30 s at 72°C, and 3 s plate read at 80°C. bacterial 16S rRNA genes were amplified for 35 cycles, where 45 cycles were used for the archaeal 16S rRNA genes. In addition, our qPCR protocol employed an annealing temperature of 57°C for archaeal 16S rRNA genes. DNA for standard curves were cloned from *E. coli* (bacterial 16S rRNA gene) or *Methanosarcina barkeri* (archaeal 16S rRNA gene), as previously described (Wojcik et al., 2018). The analysis of the data was performed with the CFX Manager software of Bio-Rad.

### Bioinformatic and statistical analysis

Sequencing raw reads were demultiplexed and adapter and quality trimmed using cutadapt v3.4 using the pair-end mode and the following parameters: -e 0.2 -q 15,15 -m 150 --discard-untrimmed (Martin, 2011). The ASVs were generated using trimmed reads and DADA2 package v1.20 (Callahan et al., 2016, p. 2) using the pooled approach with the following parameters: truncLen = c(240,200), maxN = 0, rm.phix = TRUE, compress = TRUE, multithread = TRUE, minLen = 150 with R v4.1 (R Core Team, 2018). Taxonomic assignment was done using DADA2 and SILVA database v138. Subsequently, ASVs representing chloroplasts, mitochondria and singletons were removed. Any ASV and genus presented in at least 2 out of the 5 negative controls (43 ASVs in total), or belonging to taxa considered common lab contaminants (Salter et al., 2014; Glassing et al., 2016) (44 ASVs in the rest) were removed, resulting in a dataset consisting of 552 ASVs.

Sequencing data was deposited at the EuropeanNucleotideArchive and is accessible *via* the project accession PRJEB57436.

Inter sample diversity (beta-diversity) was determined by non-metric multidimensional scaling (NMDS) using Bray–Curtis dissimilarity distances in PAST4 (Hammer et al., 2001). The ASV table was Hellinger transformed for this analysis.

The final curated ASV table was subsampled for a sequencing depth of 1,108 sequences (lowest sequence count) for alpha diversity analysis. Subsampling and subsequent alpha diversity indices calculation was conducted using the phyloseq (McMurdie and Holmes, 2013) package in R, then visualized using the ggpubr v0.4.0 (Kassambara, 2023) package in R (R Core Team, 2013).

To present the taxonomic composition of the evaluated samples, pie charts were generated using the Matplotlib (Hunter, 2007) package in Python version 3.7.3 (Van and Drake, 2009). For illustration purposes taxonomic composition was further summarized at the order and genus levels and visualized using the ggplot2 (Villanueva and Chen, 2019, p. 2) package in R.

## Results

### Geochemical and microbiological characteristics of original samples/sediments

In this manuscript we describe our efforts to enrich and characterize sediment samples recovered from the Eger Rift subsurface, with the goal to evaluate the potential for methanogenic growth under high CO_2_ conditions. The original collection of drill core sediment samples (*n* = 24) have previously been analyzed, providing insights into geochemical characteristics and microbial composition (Lipus et al., 2022).

### Methane production of enrichment cultures

**Figure 2:**
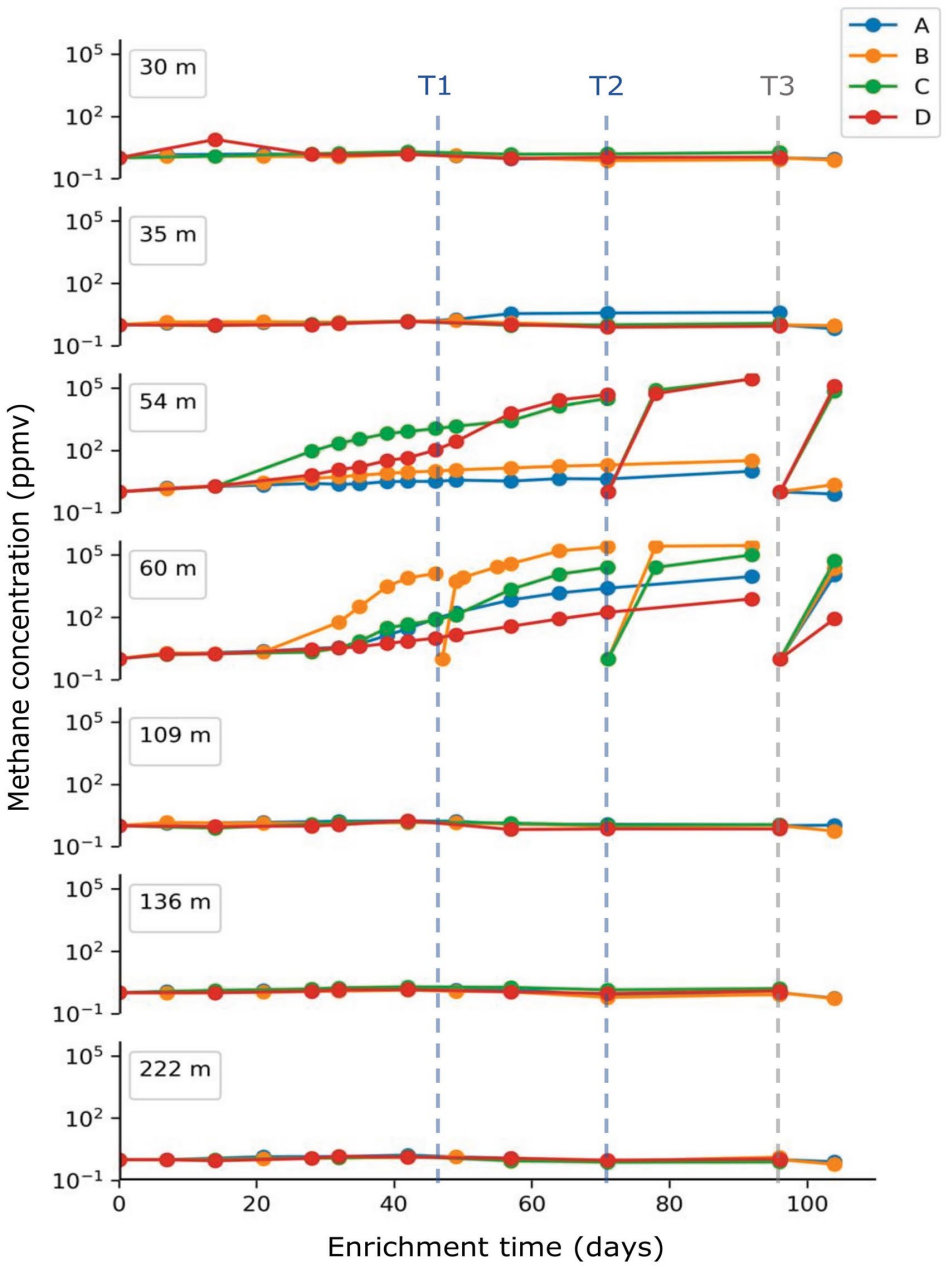
Measurement of methane concentration in the headspace of enrichment cultures. Methane concentrations were monitored every 7–10 days during the enrichment. Headspace was re-flushed with H_2_/CO_2_ mixture gas on day 49 (T1) for sample 60B, on day 71 (T2) for sample 54C, 54D, 60B, 60C and on day 96 (T3) for all replicates. Culture slurry for DNA extraction was collected from each sample replicate at T3.

Methane concentration in the headspace of each enrichment culture was monitored in approximately 1-week intervals as shown in Figure 2. Methane concentrations started to increase approximately 1 month after inoculation. Among the four enrichment replicates (A-D) started for each depth, two replicates from the 54 m sample and three from the 60 m sample were found to produce significant amounts of methane, with concentrations as high as 2.8 × 10^5^ ppmv (28%), while we observed only minor methane production (<100 ppmv) for the remaining enrichment cultures over the 3-month incubation period (Figure 1). Enrichment cultures inoculated with sediments from samples shallower than 35 m or deeper than 109 m showed no noticeable methane production with concentrations similar or lower than the air baseline value (around 2 ppmv). In samples 54C, 54D, 60A-D methane levels also rose rapidly again following subsampling for biological analyses and subsequent flushing of the headspace with fresh H_2_/CO_2_ gas mixture.

### 16S rRNA gene quantification by qPCR

**Figure 3:**
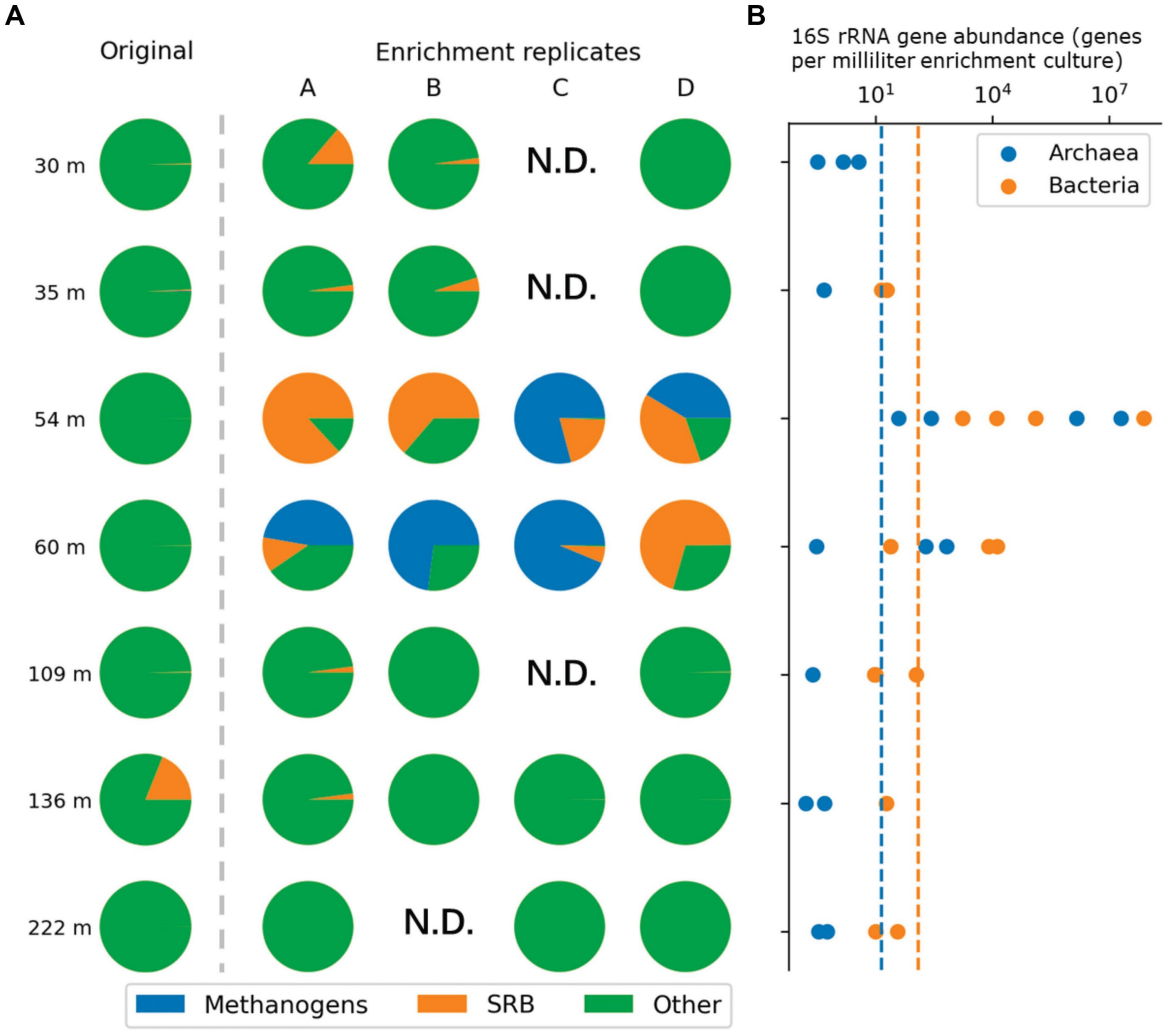
Relative abundance of methanogens and sulfate reducing bacteria in the original samples (left of the dash line) or enrichment cultures (right of the dash line) indicated through 16S rRNA gene amplicon sequencing results visualized in pie charts **(A)**. Corresponding qPCR quantification results of enrichment cultures from the same depth are depicted on the right **(B)**. Subsampling of the enrichment cultures for data shown here was carried out at T3 as shown in Figure 2.

We evaluated microbial abundance in enrichment cultures by using qPCR with primers specifically targeting either Bacteria or Archaea (Supplementary Table S1). qPCR data suggested that cultures that were started with sediment recovered from 54 m and 60 m depth were specifically enriched, as 16S rRNA gene abundance as high as 2 × 10^7^ mL^−1^ for Bacteria and 3.2 × 10^7^ mL^−1^ for Archaea were detected (Figure 3B). Likely due to low biomass qPCR measurements for the remaining enrichment cultures were below the detection limit.

### Diversity varies between active and inactive enrichment cultures

Headspace methane measurement and qPCR results suggest that the majority of enrichment cultures exhibit little or no microbial activity and harbor no significant biomass. These observations need to be taken into account when evaluating the microbial communities identified across the analyzed series of enrichments.

Despite the fact that the majority of the qPCR results are below the detection limit, we were able to extract DNA and produce 16S rRNA gene amplicon libraries for 24 of the 28 of the enrichment replicates. After curation, 16S rRNA gene sequencing generated a total of 934,292 reads, with an average sequencing depth of 28,929 sequences per sample. Data from samples 30C, 35C, 109C, 222B were discarded due to read numbers lower than 1,000. Lowest sequencing counts were observed for sample 136B with 1,108 reads and highest sequence counts were achieved for samples 60D with 182,813 reads (Supplementary Table S2).

**Figure 4:**
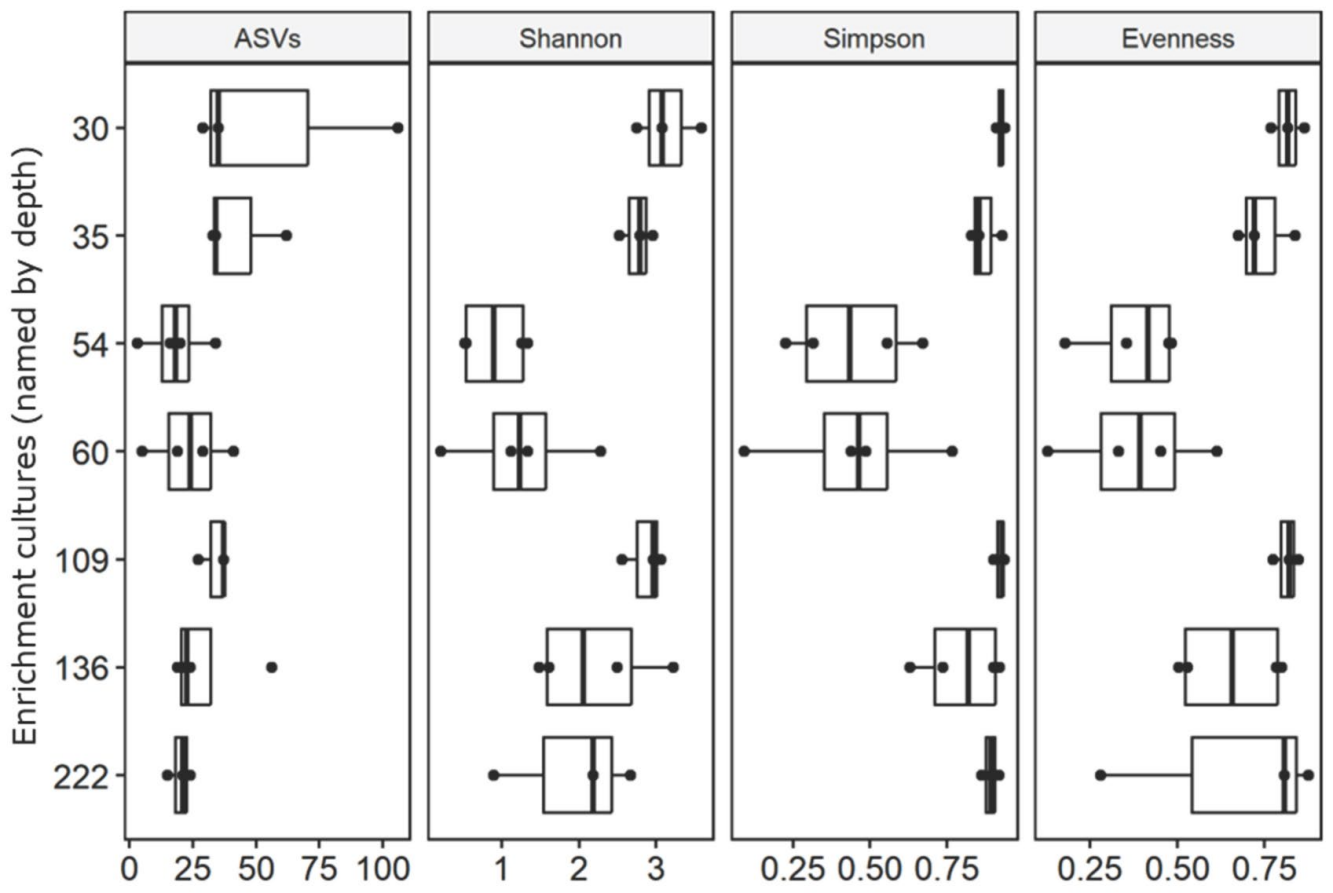
Alpha diversity indices including number of ASVs, Shannon index and Simpson index of the enrichment cultures based on curated and rarefied 16S rRNA gene amplicon sequencing dataset. Corresponding Pielou’s evenness index are shown on the right.

Differences between active and inactive enrichments were highlighted by overall diversity and differences in community structure. Active enrichments, inoculated with sediment from 54 m and 60 m depth were less diverse, between 3 and 41 ASVs were identified per 1,108 sequences. In contrast, enrichments started with deeper drill core material exhibited a greater diversity, between 15 and 106 ASVs were identified per 1,108 sequences (Figure 4, Supplementary Table S2). Similar patterns were also observed for other alpha-diversity indices including the Shannon index and the Simpson index (Figure 4, Supplementary Table S2). Pielou’s evenness index was calculated to evaluate the effect of dominant taxa on the diversity in each enrichment. Results suggested microbial communities in enrichments from samples 30 m, 35 m, 109 m, 136 m to be most even and consist of a large number of equally abundant ASVs (Figure 4, Supplementary Table S2). In contrast, analysis of evenness revealed microbial communities in enrichments from 54 m and 60 m samples to be dominated by few abundant ASVs. Non-metric multidimensional scaling further supported the differences in community structure between active and inactive enrichments, as samples from these groups clustered separately from each other on an ordination plot (Supplementary Figure S1).

### Taxonomic assessment of microbial community

**Figure 5:**
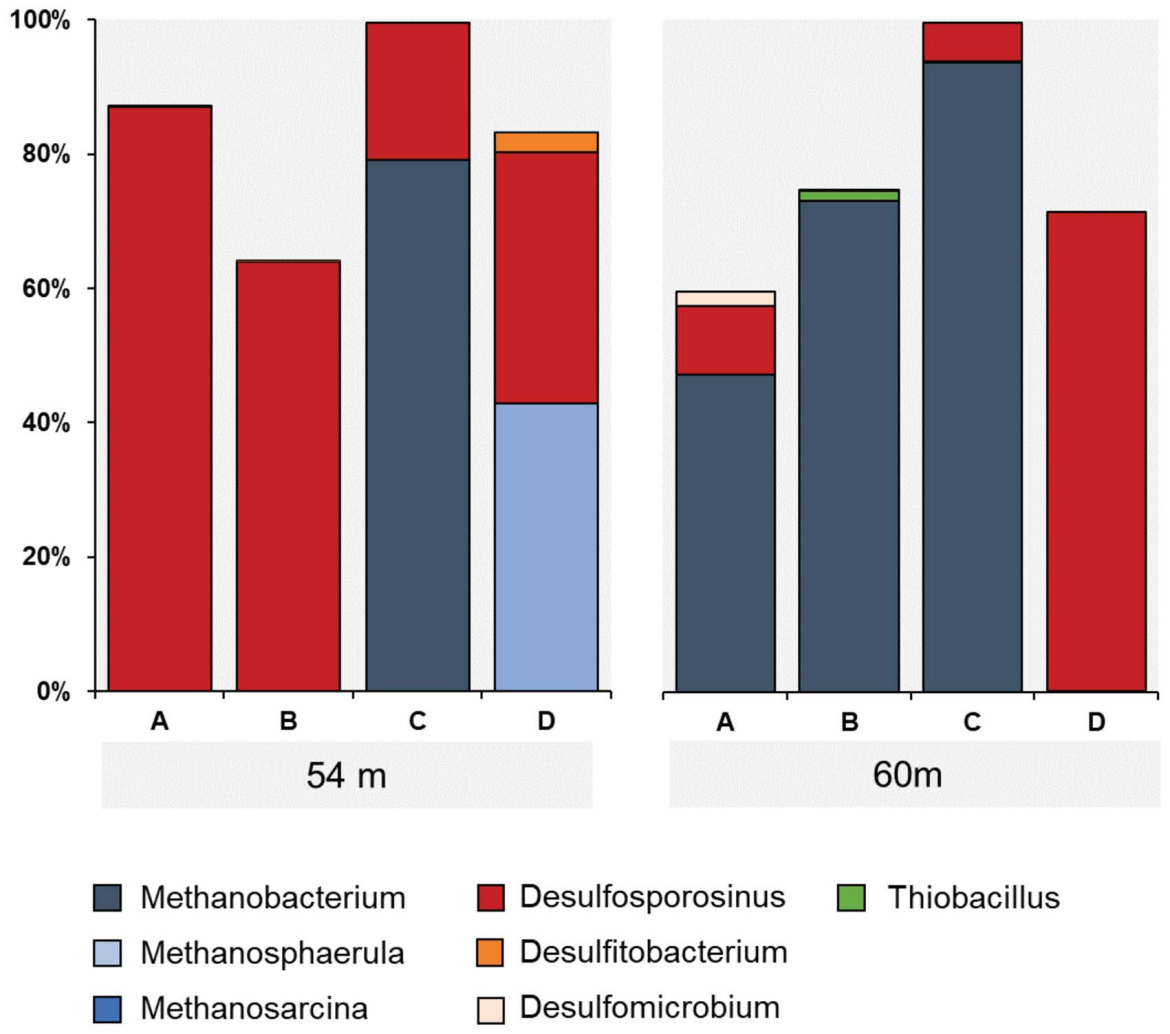
Distribution of methanogens (blue), CO_2_ (green) and sulfate utilizing (red) microbial taxa in enrichment cultures from samples 54 m and 60 m.

**Figure 6:**
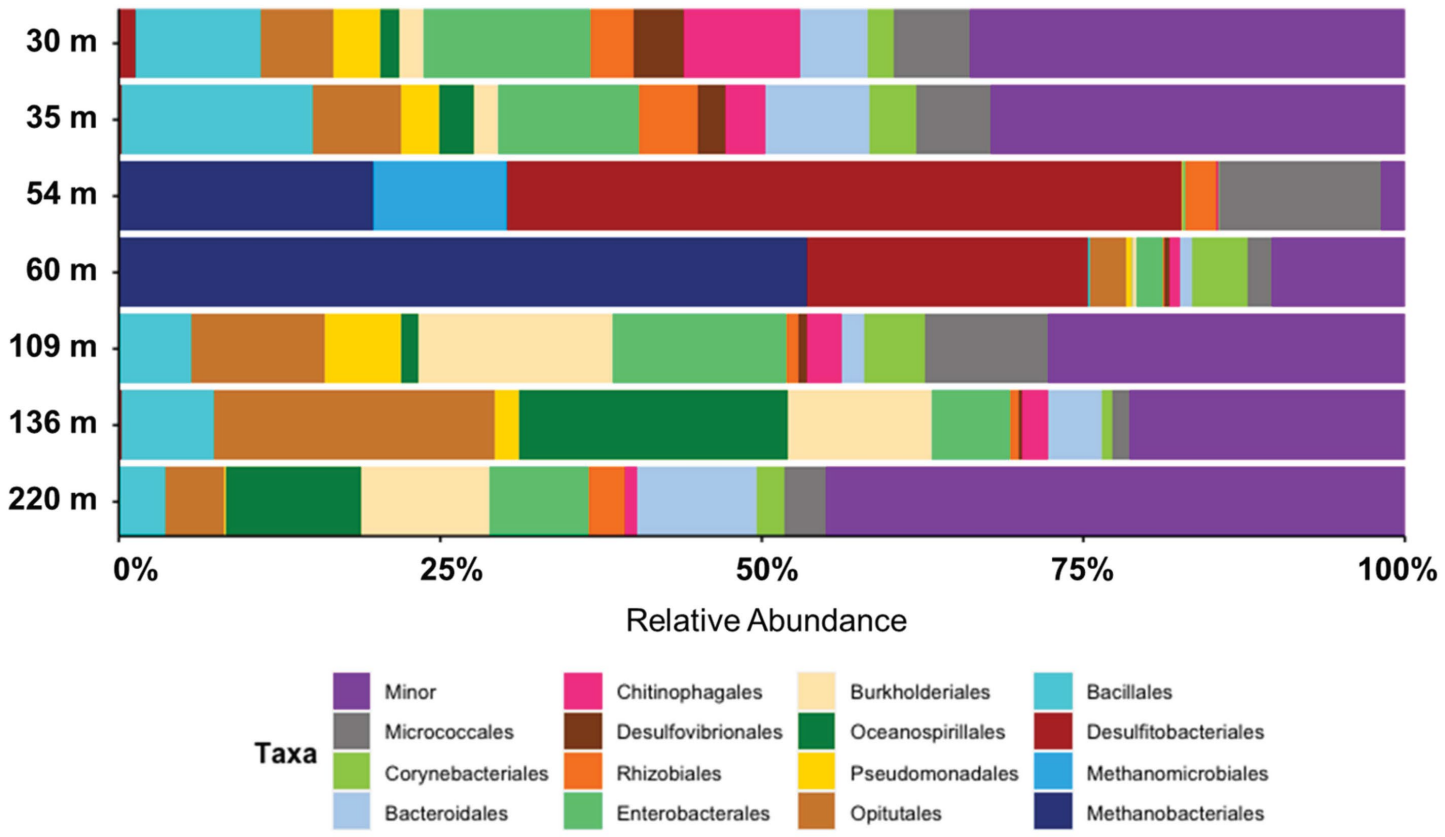
Taxonomic breakdown (top 15 orders) of microbial communities enriched from Eger Rift sediments covering seven different depths. Data shows average relative abundance from three or four biological replicates. Minor category includes all remaining microbial taxa.

Taxonomic assessment revealed microbial communities in active enrichments to be dominated by methanogenic archaea and sulfate reducing bacteria (SRB) (specifically 54C, 54D, 60A, 60B, 60C). However, even among the active enrichments we observed differences in community structure between biological replicates (Figure 3). Methanogen abundances in active methanogenic enrichments ranged from 41.4 to 93.7%, and SRB abundance ranged from less than 0.2 to 38.9% (Figure 5). Lower level taxonomic classification suggested the enriched methanogens to belong to the genera *Methanobacterium* (0.01–93.7%), *Methanosphaerula* (0–41.4%) and *Methanosarcina* (0–0.08%) (Figure 5). The majority of SRB could be classified as *Desulfosporosinus* (up to 100%), while *Desulfomicrobium* and *Desulfitobacterium* appeared in two samples, accounting for 16.8% and 7.2% of the total SRB community, respectively. None of the enriched taxa described here were found in abundances greater than 1% in the original sediment community. Other CO_2_ fixing microbes such as *Thiobacillus* were only detected in low abundances (up to 1.5%). Several minor, potentially relevant bacterial taxa, including the obligate aerobic *Pseudoarthorbacter* (up to 11%) and alkaliphilic anaerobic *Alkaliflexus* (up to 1%) could also be identified in the active enrichments (Figure 5, Figure 6). The genera *Pseudarthrobacter*, *Rhodococcus*, *Streptomyces* were also exclusively present in the enrichment cultures of samples from depths of 54 m or 60 m, suggesting they may play an important and/or specific role in the stimulation of methanogenesis upon addition of H_2_ compared to other non-methanogens, or live on the active autotrophs. *Pseudarthrobacter*, *Rhodococcus*, *Streptomyces* were also detected in the original sample community.

Low methane production and few methanogens (0-0.18%) were detected in enrichment cultures 54A, 54B, 60D, containing core samples from the same depth as other methane producing cultures. Microbial communities in these samples were dominated by Desulfosporosinus, ranging in abundance from 63.6% to 86.9%, while no other known SRB were detected. Besides Desulfosporosinus, these samples were enriched in *Pseudarthrobacter* and *Streptomyces*.

The remaining enrichments, in which little or no methane production could be measured, were dominated by heterotrophic anaerobic or obligate aerobic bacterial taxa, with the orders Bacillales (up to 16%), Opitutales (up to 23%), Pseudomonadales (up to 7%), Oceanospirillales (up to 21%), and Micrococcales (up to 13%) being specifically abundant (Figure 6). Closer taxonomic evaluation showed the genera *Diplosphaera* and *Bacillus* to be the most abundant genera (Supplementary Figure S2). In addition, sulfate reducing taxa that were highly abundant in the active enrichments were also detected in the non-methanogenic enrichments albeit at significantly lower relative abundances than those observed in the active cultures. *Desulfomicrobium* was specifically abundant in enrichments from samples 30 m (4.5%) and 35 m (2.8%), but was also detected in enrichments from deeper sediments. *Desulfosporosinus* and *Desulfitobacterium* were also detected, but at lower relative abundances (up to 1.5%). Notable was that SRB were especially abundant in replicate 30A, with 14.3% of the total microbial community. Methanogen-affiliated ASVs were generally absent from the remaining enrichments, with the exception being one replicate from 109 m, in which we detected a *Methanobacterium ASV* with a relative abundance of 0.08%.

## Discussion

Understanding the impact of CO_2_ on microbial distribution patterns and processes is essential to advance geological carbon sequestration research. While previous research efforts have provided first insights into the taxonomic and genomic diversity within natural and artificial high CO_2_ ecosystems (Oppermann et al., 2010; Martin, 2011; Beulig et al., 2015; Chen et al., 2016), including the deep biosphere of the Eger Rift (Liu et al., 2020; Lipus et al., 2022), our work aimed to specifically enrich, cultivate and describe CO_2_ utilizing microorganisms from sediment recovered from a natural high CO_2_ environment. Our data show the emergence of methanogenic archaea and sulfate reducing bacteria in selected enrichments upon addition of hydrogen, suggesting these groups of microorganisms to benefit from elevated levels of H_2_ under a constantly high CO_2_ background.

### Enrichment of methanogens

Under the given conditions, methanogens actively grew in enrichments from two of the seven sampled depths, as shown by the production of methane, elevated biomass, and increased relative abundance. Detailed classification revealed that the three enriched methanogenic taxa belong to the genera *Methanobacterium*, *Methanosphaerula* and *Methanosarcina*. It has been concluded based on the isolates that all three genera are capable of hydrogenotrophic growth (Boone, 2015; Boone and Mah, 2015; Zinder and Cadillo-Quiroz, 2016); the genus of the most abundant one, *Methanobacterium*, is documented as not being able to use methyl amine, methanol or acetate for methanogenesis (Boone, 2015). These findings indicate that the enriched methanogen community can directly utilize the provided H_2_ and CO_2_ and produce methane.

One unexpected observation is that, despite the high detection rate of methanogens throughout the original drill core samples, the active methanogen populations were not as prevalent and only enriched in cultures inoculated with samples from 54 m and 60 m depth. Moreover, at 54 and 60 m depth methanogens were not particularly abundant in the original sediments (Lipus et al., 2022). The discrepancy in enrichment growth suggests that not all natively detected methanogens are able to be stimulated by the addition of H_2_ even if CO_2_ is always abundantly available as the samples were stored in a 100% CO_2_ atmosphere. Possible explanations for these observations are that methanogens present in samples from other depths (1) are no longer viable, (2) are inhibited geochemically, (3) are outcompeted by other groups of microbes, or (4) cannot utilize H_2_ for methanogenic growth. However, close examination of the methanogenic community in the original samples suggested that most identified methanogenic taxa belong to putative obligate hydrogenotrophs, and less than 30% of the native methanogen community is composed of the metabolically versatile Methanosarcinales (Lipus et al., 2022). Methanomassiliicoccales, a group of obligate methyl-respiring methylotrophic methanogens (Nkamga and Drancourt, 2016), were only detected in two of the 36 samples with methanogen signatures. These data are against the fourth explanation. The enrichment cultures started with samples recovered from depth other than 54 m or 60 m were generally low in biomass and the identified community more diverse and similar to that in the original core sediment, thus it is also unlikely that methanogens were outcompeted for a lack of obvious growth of competitors. Alternatively, there may be additional unexplored, geochemical factors, which may have inhibited microbial growth in general and/or may have specifically impacted methanogenic growth, or methanogens in enrichments with no methane production might be no longer viable.

### Competition between methanogens and sulfate reducers

While methanogenic archaea could be enriched using sediment from 54 m and 60 m depth, methanogens were outcompeted by sulfate reducing bacteria, specifically *Desulfosporosinus* in two and one of the four replicates in enrichments from 54 m and 60 m, respectively. With sulfate concentrations as high as 200 mg/L at these depths (Figure 1D) measured through ion leaching from 5 gram of drill core sample with 25 mL MilliQ water, the sulfate concentration in the enrichment cultures would not exceed much of this value. Our results show that H_2_ utilizing sulfate reducers, like *Desulfosporosinus* can also take advantage of the unique subsurface conditions in the Eger Rift and respond to the presence of H_2_. As sulfate reduction is energetically more favorable than methanogenesis, the emergence of these lithotrophic sulfate reducers is not unexpected. Leaching experiments suggested sulfate levels in original sediments and to be in the low mM range (Lipus et al., 2022), conditions similar to those found in sulfate–methane transition zones. Although these conditions are favorable for anaerobic methane oxidizers (ANME) no ASVs belonging to these taxa were found in the original samples and enrichment cultures. As described above, analysis of original sediments suggested relative abundances as high as 15% for *Desulfosporosinus* and other SRB taxa, however these taxa were only present at very low abundances in original sediments from samples 54 m and 60 m (up to 0.3%) (Lipus et al., 2022), suggesting that they become active under the right conditions, similar to the methanogens observed here. This is especially relevant as the genus *Desulfosporosinu*s is known to contain spore formers (Lee et al., 2009; Vandieken et al., 2017), and can survive under highly acidic conditions (Alazard et al., 2010; Xu et al., 2020). In some replicates both methanogens and SRB were enriched simultaneously, probably due to relatively high H_2_ concentrations, relieving growth limitations with regard to H_2_. Sulfate reducers exclusively grew in the same enrichment cultures as the methanogenic archaea, and were slightly abundant in two replicates inoculated with 30 m deep sediment, suggesting this group of microorganisms to be similarly affected by inhibition or unviable cells across the remaining enrichment conditions.

### Covering the rare biosphere

The rare biosphere is a microbial ecological concept popularized by Sogin et al. and others (Curtis et al., 2002; Sogin et al., 2006), referring to microbes with low abundances, that still account for most of the observed phylogenetic diversity in a community and may significantly contribute to overall microbial activity. Data from our enrichment experiments suggest that both methanogenic archaea and sulfate reducing bacteria could be attributed to this group in the Eger Rift subsurface. In the majority of the original Eger Rift drill core sample methanogens were detected at low relative abundances (<1%). There were a few exceptions at depths of 30, 42 and 109 m where up to 5.5% of the microbial community were methanogens. Similarly, sulfate reducing bacteria were only identified at relatively low abundances in these samples. These groups of microorganisms may go from barely detectable during seismically quiet times, to accounting for the majority of microbial turnover during earthquake season. During quiet times with no seismic activity, we consider the subsurface of Eger Rift to be in a steady state situation with constantly elevated CO_2_ concentrations. This steady state situation is periodically disrupted by swarm earthquakes releasing H_2_, and triggering microbial growth.

Thus, our results emphasize the limitation of only using microbial census approach (i.e., just amplicon-based sequencing of native samples) to evaluate a microbial ecosystem, particularly one with low biomass, shaped by infrequent geological and geochemical events. In such a scenario a diverse pool of microbes might not be detected at all or their ecological importance is not recognized, due to their low sequencing counts. Including an additional, culture-based approach may aid in the assessment of species from the rare biosphere, and their potential to become active and more dominant once a favorable environmental change occurs. Of course, environmental factors influencing ecosystem activity, in this specific case the presence of H_2_ and CO_2_ through geogenic processes, need to be known to test such scenarios and to set up enrichment cultures with appropriate conditions.

This study clearly shows how the uneven distribution of specific microbes in different samples can lead to strong differences between biological replicates. Given that diverse physicochemical and conditions on the micrometer to millimeter scale lead to strong spatial differences in microbial community composition and density (Kallmeyer et al., 2015). Not all enrichment cultures from the same depth had similarly active methanogen populations and high percentages of methanogens among the total microbial community. Thus, sediment heterogeneity needs to be considered when examining microbial communities in subsurface samples with low biomass. In cases where specific microbes are only present in very low abundances in certain samples due to their uneven distribution, they would not be detected through DNA sequencing-based methods. Alternatively, some microorganisms might not be detected due to the challenges associated with the genomic characterization of low biomass communities (little DNA yield, and poor sequencing library quality). Such datasets are then likely only indicative of the bulk community composition, and miss less abundant taxa.

The lithology of our drill core varies greatly with depth (Figure 1C, Lipus et al., 2022), as peaty clay- and mudstone dominates the upper 80 m, followed by sandy mudstone from 80 to 100 m and weathered schist below 100 m. In this study we enriched active methanogens only from the lacustrine sediments of the Cypris formation, deposited over a coal seam. Organic carbon content of the claystone ranges between 1.8 and 6.8 wt% (Kříbek et al., 2017), providing suitable conditions for heterotrophic bacteria and methanogens. Moreover, the depositional environment (organic-rich freshwater sediments) fosters methanogenesis over other terminal electron acceptor process like sulfate reduction or metal reduction due to the lack of electron acceptors like metals or sulfate.

### Implications of enrichment results on CCS site selection and procedures

Carbon Capture and Storage (CCS), has the goal to permanently store CO_2_ in underground structures, thereby preventing it from being released into the atmosphere (Metz et al., 2005). CO_2_ is usually injected in its supercritical state, allowing more efficient usage of the available subsurface space. One major concern associated with CCS is its impact on geochemical and microbial processes occurring in the subsurface storage sites. Even under optimum conditions, a minor proportion of the injected CO_2_ will leak into the surrounding environment (Gholami et al., 2021) and potentially alternative ecosystems. Studies have shown that long-term high CO_2_ flux leads to a microbial community of low diversity and abundance, adapted to the high CO_2_ conditions (Gulliver et al., 2019). Both methanogenic archaea and sulfur cycling taxa, including autotrophic sulfur oxidizers as well as sulfate reducers may be found in natural and artificial high CO_2_ sites (Emerson et al., 2016; Freedman et al., 2017; Probst et al., 2017; Gulliver et al., 2019), posing a potential risk due to biogenic methane and sulfide production in these systems if H_2_ become available. Data from our enrichment study support this theory, showing that even if merely above the detection limit or not detectable at all, CO_2_-driven methanogenic and sulfate reducing microbial populations can emerge and become active under the right environmental conditions. Our findings specifically emphasize the need to take temporal or spatial availability of microbial substrates into account.

In our experiment, we added H_2_ to simulate a sudden increase in H_2_ concentration due to tectonic activity at greater depth. While the frequent tectonic activity would preclude the Eger Rift as a potential storage site for CO_2_, geogenic H_2_ is ubiquitous in the subsurface, at least in low concentrations. H_2_ produced by other abiotic or biotic processes, such as radiolysis of water or microbial fermentation, is not limited to unique tectonic regions and thus its potential effects on CCS projects should not be neglected.

We therefore argue that geogenic H_2_ should be taken into account when planning CCS projects. We specifically showed that H_2_ can stimulate both microbial methane production and sulfate reduction under high CO_2_ concentrations similar to those found in CCS sites.

Besides carbon sequestration, underground facilities are also used for the storage of other natural gasses, including hydrogen. Similar to CSS sites, microbial activity can have deleterious effects in these systems. Microbial activity can lower the quality of the stored gas (Buzek et al., 1994) and/or corrode the storage infrastructure (Dopffel et al., 2021). For example, SRB can actively use the ambient H_2_ and sulfate for growth, producing H_2_S that would lower the pH, lead to metal corrosion, and cause “gas souring.” This phenomenon describes accumulation of H_2_S in natural gas and is frequently observed in oil and gas wells and associated gas storage infrastructure (Vikram et al., 2016; Lipus et al., 2017; Tinker et al., 2022). Our enrichment experiments highlight the potential for sulfate reduction, even with relatively low *in-situ* sulfate concentrations and a small pool of sulfate reducers in the native sediment, thereby showing that harmful microbial processes caused by rare biosphere taxa can easily occurr in basically any type of subsurface storage facility. In summary, our study shows that simple genomic microbial investigations, such as 16S rRNA gene surveys, may be inadequate for a comprehensive evaluation of microbial potential for CCS and natural gas storage site selection. Microbes belonging to the above described rare biosphere as well as site-specific geological and geochemical characteristics, such as the geogenic production of H_2_ and the presence of sulfate, need to be taken into account when undergoing such evaluations.

Especially in the inactive samples we identified a large diversity of different types of microorganisms, which were not directly associated with methanogenesis and/or CO_2_ utilization. These types included organisms typical for anaerobic environments such as members of the Bacillales or the genus *Anaerosporobacter*, but also a variety of aerobic or moderately aerophilic heterotrophs including *Diplosphaera*, *Pseudarthrobacter*, *Rhizobium*, *Rhodococcus* or *Pseudomonas.* Throughout our experiment we maintained strict anaerobic conditions through the addition of cysteine and the redox potential was permanently kept below −110 mV, based the colorless appearance of the redox indicator resazurin in the medium (Ruseler-van Embden and Both-Patoir, 1985). The most likely explanation for the presence of aerobic taxa in our enrichments is that they belong to the original low biomass community found in the subsurface sediments and rocks used for our experiments. Several of the identified taxa match with the communities previously described in Eger Rift subsurface sediments (Liu et al., 2020; Lipus et al., 2022). While we used strict contamination controls it is also possible that some of these taxa may come from external sources, which can be an issue, especially when working with low biomass communities. Nevertheless, additional work may be required to fully elucidate the occurrence of these aerobes under anaerobic conditions.

## Data availability statement

The datasets presented in this study can be found online in the European Nucleotide Archive (ENA) at https://www.ebi.ac.uk/ena under accession number PRJEB57436.

## Author contributions

JK, DL, and ZJ designed the experiment. ZJ, MS, and OB carried out the enrichment cultures. ZJ extracted DNA and prepared sequencing libraries. AB provided bioinformatic analyses. JK and DL did the field work. RB provided geologic and stratigraphic information. ZJ and DL wrote the manuscript with input from all other co-authors. All authors contributed to the article and approved the submitted version.

## Funding

ZJ is funded by the China Scholarship Council. DL was funded by the German Research Foundation (DFG) ICDP Priority Program (grant number for DFG fellowship: 419459207). Drilling was part of the “Drilling the Eger Rift” project of the International Scientific Drilling Program (ICDP).

## Conflict of interest

The authors declare that the research was conducted in the absence of any commercial or financial relationships that could be construed as a potential conflict of interest.

## Publisher’s note

All claims expressed in this article are solely those of the authors and do not necessarily represent those of their affiliated organizations, or those of the publisher, the editors and the reviewers. Any product that may be evaluated in this article, or claim that may be made by its manufacturer, is not guaranteed or endorsed by the publisher.

## Supplementary material

SUPPLEMENTARY TABLE 1 Genus level taxonomy table.

SUPPLEMENTARY DATA SHEET 1 List of primers pairs used in this study; statistical data of the library; NMDS plot of the distance between samples; Top 20 Genus level taxonomic breakdown of the microbial community in enrichments cultures.
