# Supplementary material for: Enrichment of rare methanogenic Archaea shows their important ecological role in natural high-CO2 terrestrial subsurface environments
Source: Front Microbiol. 2023 May 24;14:1105259. doi: 10.3389/fmicb.2023.1105259 (PMC10246774; doi:10.3389/fmicb.2023.1105259)
Supplement: SUPPLEMENTARY DATA SHEET 1 — List of primers pairs used in this study; statistical data of the library; NMDS plot of the distance between samples; Top 20 Genus level taxonomic breakdown of the microbial community in enrichments cultures. [file Data_Sheet_1.docx]

| Primer | Sequence (5' - 3') |
| --- | --- |
| Uni515-F | GTGYCAGCMGCCGCGGTAA |
| Uni806-R | GGACTACNVGGGTWTCTAAT |
| Eub341-F | CCT ACG GGA GGC AGC AG |
| Eub534-R | ATT ACC GCG GCT GCT GG |
| Parch340-F | CCC TAY GGG GYG CAS CAG |
| Arch1000-R | GGC CAT GCA CYW CYT CTC |

Table S1. Primers used in this study.

|  | Library size | ASVs | Shannon | Simpson | Evenness |
| --- | --- | --- | --- | --- | --- |
| 30a | 20456 | 35 | 3.0709982 | 0.93808892 | 0.8637686513 |
| 30b | 57655 | 106 | 3.5850519 | 0.92702726 | 0.768757097 |
| 30d | 1793 | 29 | 2.7493353 | 0.90945079 | 0.8164816633 |
| 35a | 30485 | 62 | 2.7851817 | 0.82879029 | 0.6748463801 |
| 35b | 4369 | 34 | 2.9537534 | 0.92951655 | 0.8376209351 |
| 35d | 43393 | 33 | 2.5239212 | 0.85397633 | 0.721840624 |
| 54a | 134025 | 20 | 0.540476 | 0.22674119 | 0.1804153211 |
| 54b | 101392 | 34 | 1.2547951 | 0.55507044 | 0.3558329023 |
| 54c | 122374 | 3 | 0.5228803 | 0.31547883 | 0.4759461599 |
| 54d | 182815 | 16 | 1.339335 | 0.67189068 | 0.4830629906 |
| 60a | 7794 | 41 | 2.2815501 | 0.76629436 | 0.6143815332 |
| 60b | 137917 | 29 | 1.1221343 | 0.43992004 | 0.3332449409 |
| 60c | 55325 | 5 | 0.2086396 | 0.09141915 | 0.1296350722 |
| 60d | 10178 | 19 | 1.3326993 | 0.48618352 | 0.4526156967 |
| 109a | 2637 | 37 | 3.0572776 | 0.93516793 | 0.8466760181 |
| 109b | 33541 | 37 | 2.9592755 | 0.92875575 | 0.819535523 |
| 109d | 1355 | 27 | 2.5543804 | 0.90097616 | 0.7750324133 |
| 136a | 3427 | 56 | 3.2187803 | 0.92106798 | 0.7996271003 |
| 136b | 1108 | 21 | 1.6153683 | 0.7358691 | 0.5305818344 |
| 136c | 6885 | 24 | 2.4927845 | 0.90213772 | 0.7843745365 |
| 136d | 2817 | 19 | 1.4846201 | 0.62963319 | 0.5042115359 |
| 222a | 1265 | 24 | 0.89260905 | 0.89260905 | 0.280866561 |
| 222c | 1139 | 21 | 2.6673559 | 0.91909839 | 0.8761163547 |
| 222d | 2160 | 15 | 2.1853312 | 0.86113138 | 0.8069758822 |

Table S2. Library size, alpha diversity indices and Pielou’s evenness index of the enrichment cultures’ microbial communities.


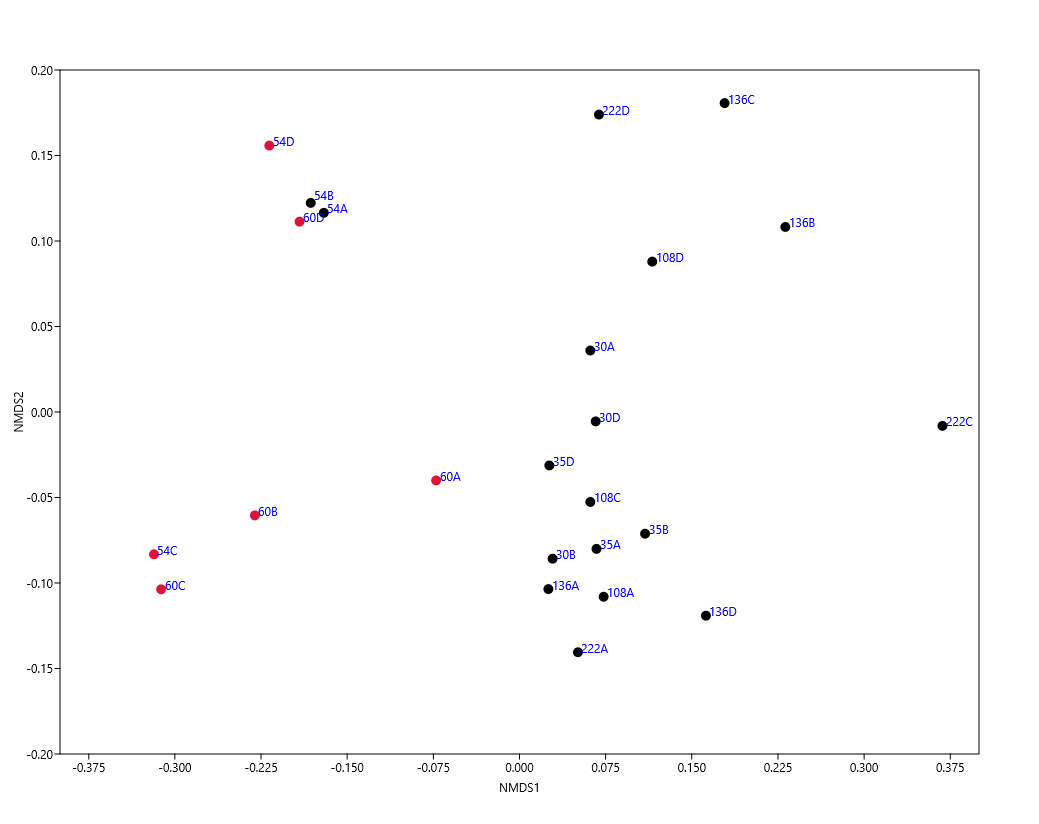


Figure S1. Non-metric multidimensional scaling shows the inter-sample diversity.


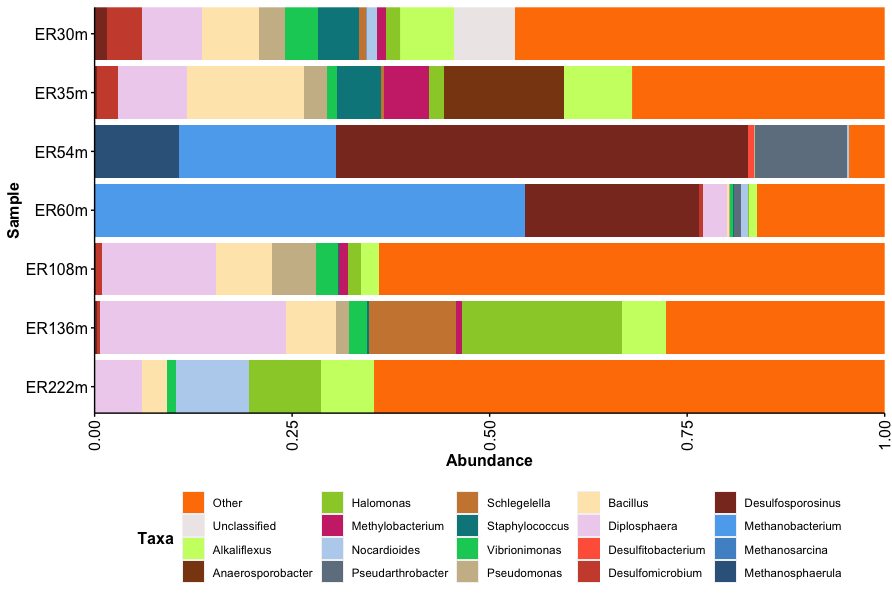


Figure S2. Top 20 Genus level taxonomic breakdown of the microbial community in enrichments from Eger Rift sediment across seven different depths.
